# Comparison of test performance of two commonly used multiplex assays to measure micronutrient and inflammatory markers in serum: results from a survey among pregnant women in South Africa

**DOI:** 10.1017/S0007114523001782

**Published:** 2024-01-28

**Authors:** Tsitsi Letwin Chimhashu, Hans Verhoef, Elizabeth A. Symington, Lizelle Zandberg, Jeannine Baumgartner, Linda Malan, Cornelius Marius Smuts, Edith J. M. Feskens, Alida Melse-Boonstra

**Affiliations:** 1 Division of Human Nutrition and Health, Wageningen University & Research, Wageningen, The Netherlands; 2 Centre of Excellence for Nutrition, North-West University, Potchefstroom, South Africa; 3 Department of Life and Consumer Sciences, University of South Africa, Johannesburg, South Africa; 4 Department of Nutritional Sciences, King’s College, London, UK

**Keywords:** *α*-1-acid glycoprotein, C-reactive protein, Ferritin, Quansys 7-Plex assay, Retinol-binding protein, Soluble transferrin receptor

## Abstract

The combined sandwich-ELISA (s-ELISA; VitMin Lab, Germany) and the Quansys Q-Plex™ Human Micronutrient Array (7-Plex) are multiplex serum assays that are used to assess population micronutrient status in low-income countries. We aimed to compare the agreement of five analytes, *α*-1-acid glycoprotein (AGP), C-reactive protein (CRP), ferritin, retinol-binding protein 4 (RBP4) and soluble transferrin receptor (sTfR) as measured by the 7-Plex and the s-ELISA. Serum samples were collected between March 2016 and December 2017. Pregnant women (*n* 249) were recruited at primary healthcare clinics in Johannesburg, and serum samples were collected between March 2016 and December 2017. Agreement between continuous measurements was assessed by Bland–Altman plots and concordance measures. Agreement in classifications of deficiency or inflammation was assessed by Cohen’s kappa. Strong correlations (r > 0·80) were observed between the 7-Plex and s-ELISA for CRP and ferritin. Except for CRP, the 7-Plex assay gave consistently higher measurements than the s-ELISA. With the exception of CRP (Lin’s ρ = 0·92), there was poor agreement between the two assays, with Lin’s ρ < 0·90. Discrepancies of test results difference between methods increased as the serum concentrations rose. Cohen’s kappa for all the five analytes was < 0·81 and ranged from slight agreement (vitamin A deficiency) to substantial (inflammation and Fe deficiency) agreement. The 7-Plex 1.0 is a research and or surveillance tool with potential for use in low-resource laboratories but cannot be used interchangeably with the s-ELISA. Further optimising and validation is required to establish its interchangeability with other validated methods.

Public health programmes in low- and middle-income countries urgently need accurate, reproducible and cost-efficient methods to assess micronutrient status, with a view that these methods can be used to identify populations at risk, to determine the appropriate interventions and to monitor programme impacts. In low-resource settings, representative population data on micronutrient status remain scarce^(1)^. A huge barrier to this is a lack of accurate, affordable and fast laboratory surveillance methods that utilise a small blood sample volume to effectively assess a population’s prevalence and severity of micronutrient deficiencies. The VitMin Lab combined sandwich-ELISA (s-ELISA)^(2)^ and the Quansys 5-Plex (2014) and 7-Plex (2017)^(3,4)^ can measure several biomarkers in a single run, using only a small blood sample volume.

The s-ELISA was developed by Dr Juergen Erhardt of the VitMin Lab (Willstätt, Germany) and has been widely used in studies and national surveys for micronutrient status determination^(3–7)^. It has scored well in several critical assessments^(8)^. The s-ELISA simultaneously measures three micronutrient markers and two inflammatory markers in a single small volume of sample (5 µl) and at a low cost: *α*-1-acid glycoprotein (AGP), C-reactive protein (CRP), ferritin, retinol-binding protein 4 (RBP) and soluble transferrin receptor (sTfR). The s-ELISA assay was shown to have good agreement with the ferritin RIA from Bio-Rad Laboratories (*n* 44), the sTfR ELISA assay from Ramco Laboratories (*n* 119) and the CRP ELISA assay from Immuno-Biological Laboratories Inc. (*n* 17)^(2)^. Furthermore, the s-ELISA assays produced mostly comparable results to the Roche reference-type assays for AGP, CRP and ferritin^(9)^.

The Quansys 5-Plex (AGP, CRP, ferritin, RBP and sTfR) and 7-Plex (2017) assay also concurrently measures five or seven analytes: AGP, CRP, ferritin, RBP, sTfR, histidine-rich protein II (HRP2; produced by malaria-causing *Plasmodium falciparum* parasites) and thyroglobulin in a single sample aliquot (10 µl). Researchers have described the Quansys machine as a non-diagnostic research tool that offers assays that are accurate, easy-to-use, reliable and scalable. The Q-Plex assay is thought to have the potential to overturn undesirable logistical hurdles such as international shipping of samples for analysis and sometimes restrictive sample export/import legislation^(3)^.

Over the years, researchers have compared the 7-Plex performance to that of the s-ELISA. Good agreement between a newly developed method to an established one is essential to determine interchangeability or replacement of an established method with a more advanced, faster and/or less expensive newer method^(10)^. Poor agreement between methods may lead to over- and or underestimation of deficiency prevalence estimates that undermine appropriate policy development and effective targeting of national interventions^(11)^. Contrasting results, that is, good^(3,4,12)^ and poor^(7)^ agreement between the 7-Plex and s-ELISA from various studies, have led researchers to conclude that further studies are required to evaluate the validity of the Q-Plex assays against well-established methods. Furthermore, uncertainties with the precision of the 7-Plex may be of interest to most researchers in low-resource settings. These may be a result of blood sample type, laboratory equipment, experience of technicians and laboratory conditions such as ambient temperature and effect of freeze–thaw cycles. In this study, we therefore aimed to compare the level of agreement between the Quansys 7-Plex and s-ELISA array results for AGP, CRP, ferritin, RBP and sTfR with the use of blood serum samples from pregnant women living in South Africa.

## Subjects and methodology

### Ethical considerations

Written informed consent was obtained from all the women at the first visit before data collection. Ethical approval was obtained from the Human Research Ethics Committees of the North-West University, Potchefstroom (NWU-00186-15-A1 and NWU-00456-19-A1) and the University of the Witwatersrand, Johannesburg (M150968 and M161045). Approval was also given by The Gauteng Health Department, City of Johannesburg District Research Committee and Clinical Manager of Rahima Moosa Mother and Child Hospital (RMMCH). The Nutrition during Pregnancy and Early Development (NuPED) study was conducted according to the guidelines laid down in the Declaration of Helsinki.

### Study design, setting and participants

For the purpose of the current study, we used samples and data collected from the NuPED study. The NuPED study was a prospective cohort study designed to follow women during pregnancy and their infants until 12 months of age. Pregnant women were recruited at primary healthcare clinics in Johannesburg, whilst data and samples were collected at RMMCH between March 2016 and December 2017 in Johannesburg, South Africa^(13)^.

Pregnant women (*n* 250) were included in the study if aged 18–39 years, < 18 weeks of gestation with singleton pregnancies, proficient in a local language, born in either South Africa or a neighbouring country (Botswana, Lesotho, Zimbabwe), and resided in Johannesburg for at least 12 months. Women were excluded if they reported illicit drug use, smoked or were diagnosed with a non-communicable disease such as hypercholesterolemia and hypertension diabetes or renal disease. They were also excluded when reporting to underlying and or serious illnesses such as tuberculosis, hepatitis or cancer. HIV status was not considered an exclusion criteria.

### Blood collection and storage

Venous blood was drawn into 6-ml serum separator tubes (gold tiger top tube BD Vacutainer) from the antecubital vein, centrifuged and processed within 1 h after blood draw. Serum was stored at −20°C; for a maximum of 14 d until transportation on dry ice (–80°C;) to the Centre of Excellence for Nutrition laboratories in Potchefstroom, South Africa, for storage at until analysed by Quansys 7-Plex (2018). Thereafter, frozen samples were shipped on dry ice to Wageningen University, Wageningen, the Netherlands (July 2020), where they were stored at −80°C;, thawed for s-ELISA sample preparation, refrozen and shipped on dry ice to the VitMin Lab, Willstaett, Germany (May 2021).

### Description of the ELISA methods

Q-Plex Human Micronutrient (7-Plex) kits (Quansys Biosciences) were used according to kit instructions. Full details on the Quansys Q-Plex Human Micronutrient ELISA can also be found elsewhere^(3,4)^. In our study, pooled samples were utilised to assess quality control parameters during analysis. These pooled samples served as control samples and were measured in duplicate for accuracy.

Details on the s-ELISA assay can be found elsewhere^(2)^. Capture antibodies used were ferritin (Code A0133, Dako), sTfR (Cat. No 4Tr26; Clone 23D10, Hytest), RBP (Code A0040, Dako) and CRP (Code A0073, Dako). Detection antibodies were anti-ferritin-horseradish peroxidase (Code P0145, Dako), anti-sTfR-horseradish peroxidase (Cat. No. 4Tr26-c; Clone 13E4, Hytest), anti-RBP-anti-ferritin-horseradish peroxidase (Code P0304, Dako) and anti-CRP-anti-ferritin-horseradish peroxidase^(2)^. Two calibration curves were used for each analyte to cover a wider range (online Supplementary Fig. 1). Samples were measured in duplicate. A serum quality control sample with a medium content of the five proteins was used to minimise the differences between plates and different measurement days on each 384-well plate at ten different positions. The absorption of the study samples was adjusted based on the mean absorption of this quality control. Additionally, on each plate, a quality control from Biorad (Liqicheck Immunology Control) with low, medium and high content of the five proteins was used to check the calibration curve in the low, medium and high range. The absolute values of all these quality controls were compared with results from The Vitamin A Laboratory-External Quality Assessment (VITAL-EQA). The VITAL-EQA is a quality assurance programme that was set up by the CDC in 2003 to standardise the measurements of serum vitamins and micronutrient in international studies^(14)^.

### Definitions

Commonly used classification methods in literature were used to estimate inflammation or deficiency between the two methods. Fe deficiency in the absence of inflammation was defined as serum ferritin concentration < 15 µg/l^(15)^. Since it was measured with a Ramco kit, Fe deficiency erythropoiesis was defined as sTfR > 8·3 mg/l. Elevated CRP and AGP were defined as concentrations > 5 mg/l and > 1 g/l^(16)^, respectively. Inflammatory status was defined as no inflammation (CRP concentration ≤ 5 mg/l and AGP concentration ≤ 1 g/l) *v.* any inflammation (elevated CRP and/or AGP). The internal correction factor (CF) approach proposed by Thurnham *et al.* (2012) was used to adjust Fe and vitamin A indicators for inflammation before categorising them into one of the four categories: (1) reference (CRP concentration ≤ 5 mg/l and AGP concentration ≤ 1 g/l); (2) incubation (CRP concentration > 5 mg/l and AGP concentration ≤ 1 g/l); (3) early convalescence (CRP concentration > 5 mg/l and AGP concentration > 1 g/l); and (4) late convalescence (CRP concentration ≤ 5 mg/l and concentration AGP > 1 g/l)^(15)^. With the assumption that RBP occurs in a 1:1 ratio with retinol with no variability in this ratio, vitamin A deficiency was defined as RBP < 0·70 µmol/l^(17,18)^.

### Statistical analysis

Stata software version 16 (StataCorp LLC.) and SPSS version 26 (IBM Corp) was used for analysis. As all the outcome variables were non-normally distributed, analyte concentrations are described as median and 25th–75th percentile. All analyte concentrations were unadjusted for inflammation.

Several statistical evaluation measures were used to compare the reproducibility of results between the 7-Plex and s-ELISA. First, after natural log-transformation, to quantify the extent of linearity between the two measurements, correlations between the two methods were determined by Pearson’s correlation coefficients (*r*) and scatterplots with the line of identity. Second, Bland–Altman analysis (difference plots and statistics^(19)^) were used to assess agreement between analyte measurement results from the two assays. We did this because high *r* correlation and scatter of points lying near the line of identity do not automatically imply that there is good agreement between the two methods^(20)^. To address the relationship between the non-constant difference, or the assumption of homoscedastic variance (non-constant variance) violations, we produced Brand–Altman plots again with log-transformed variables, and with regression lines to indicate potential trends in the relationship between the differences and the magnitude of the measurements^(10)^. Bias was defined as the difference in log-transformed means of the two methods’ measures. Thereafter, we back-transformed the bias to produce a geometric mean and geometric sd. The geometric sd is a dimensionless, multiplicative factor such that dividing or multiplication of the geometric mean by this ratio indicates a variation that is equivalent to subtraction or addition of 1 sd on a log-transformed scale. Limits of agreement (LoA) derived from log-transformed data were also back-transformed to yield limits for the ratio of actual measurements^(21)^. The LoA was calculated as the mean difference ± 1·96 sd of the differences, and it provides a range where 95 % of the differences (when normally distributed) between the two methods should fall^(10,20)^. Furthermore, the bias and agreement limits of CI can determine the sampling error in relation to the dimension of the sample^(20)^. The level of agreement and disagreement of the same variable by two instruments on a continuous scale was determined by log-transformed derived Lin’s concordance coefficient (ρ). The Lin coefficient combines measures of both precision and accuracy to determine how meaningful the distance from the line of perfect concordance. This 45° line quantifies the dis(agreement) between the set of analytes that were measured by two different assays (see refs. 22 and 23 for further details). We interpreted *P* < 0·90 as an indication of poor agreement, and 0·90–0·94, 0·95–0·99 and > 0·99 as moderate, substantial and almost perfect levels of agreement, respectively^(24)^.

Lastly, to evaluate agreement in categorical test results, we used Cohen’s kappa coefficient (κ) with its 95 % confidence and percent agreement interval. The kappa coefficient provides the ability of the two assays to define and classify deficiency and inflammation the same, adjusted for how often they agree by chance. The benchmark scale was used to estimate the kappa statistics: < 0·00 was interpreted as poor, 0·00–0·20 as slight; 0·21–0·40 as fair; 0·41–0·60 as moderate; 0·61–0·80 as good and ≥ 0·81 as very good^(25)^.

## Results

The inter-assay CV for the control sample from the 7-Plex analysis were as follows: AGP 11·6 %, CRP 21·1 %, ferritin 14·0 %, RBP 10·9 % and sTfR 25·5 %. Whilst the inter-assay CV for the control sample were from the s-ELISA analysis were AGP 8·1 %, CRP 5·8 %, ferritin 2·3 %, RBP 3·6 % and sTfR 3·6 %.

One subject had no sample analysed for the s-ELISA and was therefore removed from the 7-Plex analysis, leaving 249 data points for comparison of AGP, CRP, ferritin and RBP measurements. For sTfR, we excluded a further five values of measurement for the s-ELISA because they were below the limit of quantification, and one 7-Plex outlier (7-Plex: 45·8 mg/l; s-ELISA: 3·60 mg/l) that was highly influential in determining the slope of agreement in the Bland–Altman plot. Thus, the final sample size was 243.

Table 1 shows the characteristics of the study population. The mean age was 27 years, and most women were in the second trimester of pregnancy. The distributions of analyte concentrations as measured by the 7-Plex and s-ELISA assays are presented in Table 2. The prevalence of inflammatory status and Fe deficiency was similar between the two methods. However, prevalence estimates for vitamin A deficiency (5 % *v*. 0·8 %) differed substantially between the two methods, as also indicated by the corresponding κ values and % agreement presented in Table 3. Overall, using Cohen’s kappa, inter-definitional agreement between the two assays was slight to substantial (Table 3), with inflammation definitions having substantial agreements across three different evaluation approaches. The percentage of pregnant women classified differently between the two assays was 8 % for ID, 11 % for elevated CRP, 13 % for elevated AGP, 11 % for Fe-deficient erythropoiesis and 39 % for vitamin A deficiency.

**Table 1.** Descriptive characteristics of study population (*n* 249)

	Values	
	Median	25th–75th percentile
Age (years)	27	24·0–32·0
Gestational age, by ultrasound (wks)	14	12·0–16·0
Body weight (kg)	67·5	58·0–77·8
BMI (kg/m^2^)	26·3	23·0–30·6
MUAC (cm)	29·9	27·2–33·1
	*n*	%
HIV-positive	64	25·7

MUAC, mid-upper arm circumference.

**Table 2.** Micronutrient and inflammatory marker concentrations as measured by 7-Plex and s-ELISA

	7-Plex		s-ELISA	
	Median	25th–75th percentile	Median	25th–75th percentile
AGP (g/l)	0·75	0·63–0·86	0·69	0·58–0·83
CRP (mg/l)	6·48	3·08–14·36	6·92	3·12–13·1
Ferritin (µg/l)	54·4	28·4–116·4	49·1	26·7–79·0
RBP (µmol/l)	1·54	1·27–1·80	1·11	0·89–1·41
sTfR (mg/l)	4·82	3·77–6·61	4·68	3·87–6·03
	*n*	%	*n*	%
AGP > 1 g/l	28	11·2	24	9·60
CRP > 5 mg/l	148	59·4	155	62·2
Ferritin < 15 µg/l	32	12·9	35	14·1
RBP < 0·70 µmol/l	2	0·80	14	5·60
sTfR > 8·3 mg/l	38	15·3	23	9·40

AGP, *α*-1-acid glycoprotein; CRP, C-reactive protein; RBP, retinol-binding protein; sTfR, soluble transferrin receptor.

AGP, CRP, ferritin, RBP (*n* 249), and sTfR (*n* 243; see text).

**Table 3.** Agreement between 7-Plex and s-ELISA in classification of inflammation or deficiency categories

	*n*	% Agreement	Kappa, κ	95 % CI
Serum AGP concentration > 1 g/l	249	87·2	0·31	0·13, 0·50
Serum CRP concentration > 5 g/l	249	89·2	0·77	0·69, 0·85
Inflammatory status*	249	87·6	0·74	0·65, 0·82
Categorical correction factor†	249	78·2	0·62	0·54, 0·71
Fe deficiency‡	249	91·6	0·64	0·50, 0·78
Fe-deficient erythropoiesis§	243||	88·5	0·47	0·31, 0·63
Vitamin A deficiency¶	249	60·6	0·12	0·05, 0·19

s-ELISA, sandwich-ELISA; AGP, *α*-1-acid glycoprotein; CRP, C-reactive protein; CF, correction factor.

* Inflammatory status was defined as no inflammation (serum concentration of both CRP ≤ 5 mg/l and AGP ≤ 1 g/l) *v*. any inflammation (elevated CRP and/or AGP).

† Categorical CF approach as proposed by Thurnham et al.^(15)^ divided inflammation into four categories: (a) reference (CRP concentration ≤ 5 mg/l and AGP concentration ≤ 1 g/l); (b) incubation (CRP concentration >5 mg/l and AGP concentration ≤ 1 g/l); (c) early convalescence (CRP concentration >5 mg/l and AGP concentration > 1 g/l); and (d) late convalescence (CRP concentration ≤ 5 mg/l and AGP concentration > 1 g/l.

‡ Fe deficiency: serum ferritin concentration < 15 µg/l.

§ Fe-deficient erythropoiesis: serum-soluble transferrin concentration > 8·3 mg/l.

|| Six values were excluded (see text).

¶ Vitamin A deficiency: serum retinol-binding protein concentration < 0·70 µmol/l.

**Fig. 1.**
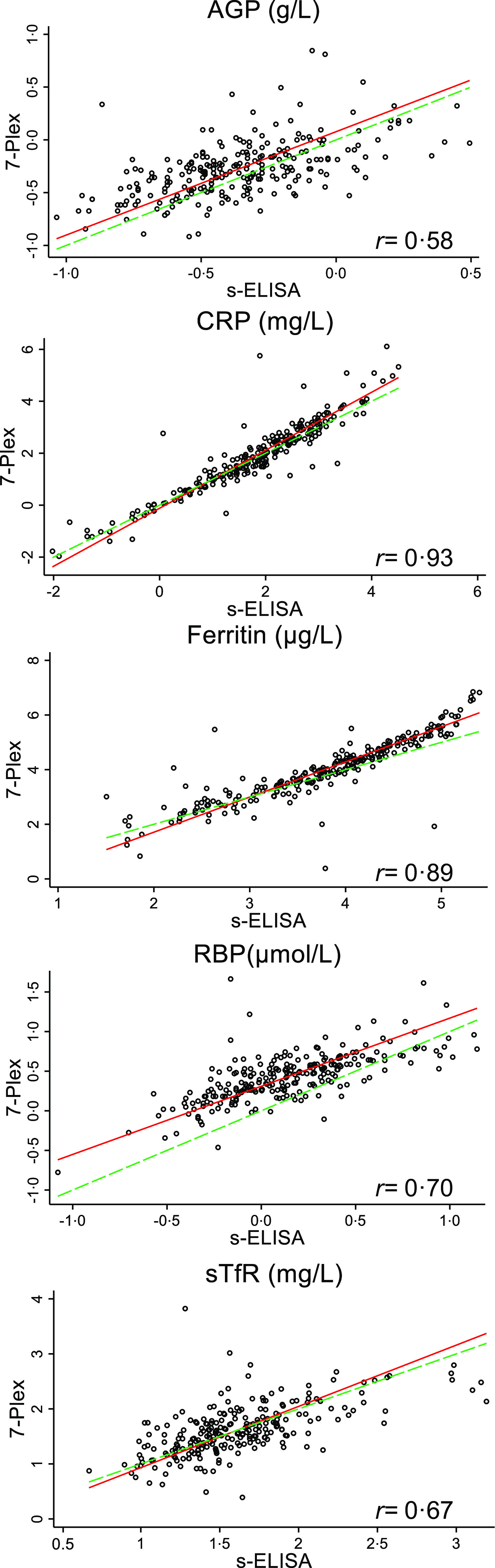
Comparison of serum concentrations of selected analytes measured by 7-Plex *v*. the s-ELISA methods. All variables are in log scale. AGP, CRP, ferritin, RBP (*n* 249) and sTfR (*n* 243; see text). Solid red line: prediction line; dotted green line: line of identity (perfect concordance). AGP, 1-acid-glycoprotein; CRP, C-reactive protein; RBP, retinol-binding protein; sTfR, soluble transferrin receptor.

Scatter graphs based on log-transformed values showing correlations between the two methods are presented in Fig. 1. The correlations between the 7-Plex and s-ELISA results were moderate for AGP (*r* = 0·58), RBP (*r* = 0·70) and sTfR (*r* = 0·67) and strong for CRP (*r* = 0·93) and ferritin (*r* = 0·89).

Bias, LoA, and correlation coefficients of AGP, CRP, ferritin, sTfR and RBP concentrations comparing the 7-Plex and the s-ELISA assays are described in Table 4. For all the analytes, the log-transformed constant variance model was selected. Geometric mean values obtained by 7-Plex were 9 %–34 % higher than values obtained by s-ELISA assays. We observed moderate concordance for CRP (Lin’s ρ = 0·92), but poor agreement for AGP, ferritin, RBP and sTfR. For all the biomarkers assessed, the 7-Plex produced higher values than the s-ELISA, with the largest difference in analytes measured being largest for ferritin readings.

**Table 4.** Performance indicators of 7-Plex compared with s-ELISA for measurement of serum ferritin, sTfR, AGP, CRP and RBP concentrations†

	Original scale data *P*	Ln-scale data *P*	Original scale data *P*	Ln-scale data *P*	Pearson’s correlation coefficient§	Bias||		Lin’s concordance§		LoA||	% Outside LoA	
Parameter	Non-constant difference‡		Non-constant variance§		r	Mean	sd	rho	95 % CI	95 % CI	*n*	%
AGP	0·004	0·688	0·995	0·115	0·58	1·09	1·28	0·56	0·47, 0·64	0·67, 1·78	12	4·82
CRP	< 0·001	0·027	0·003	0·992	0·93	1·09	1·65	0·92	0·90, 0·94	0·41, 2·91	11	4·42
Ferritin	< 0·001	< 0·001	0·991	0·999	0·89	1·25	1·70	0·83	0·80, 0·87	0·44, 3·54	11	4·42
RBP	0·010	0·001	< 0·001	0·994	0·70	1·34	1·30	0·50	0·43, 0·57	0·80, 2·23	10	4·02
sTfR*	0·004	0·211	0·998	0·998	0·67	0·99	1·42	0·67	0·60, 0·74	0·49, 1·98	15	6·17

s-ELISA, sandwich-ELISA; sTfR, soluble transferrin receptor; AGP, *α*-1-acid glycoprotein; CRP, C-reactive protein; RBP, retinol-binding protein; LoA, limits of agreement.

* sTfR (*n* 243) (six values excluded as they were outside of the limit of quantification range or they were influential outliers; see text).

† AGP, CRP, ferritin, RBP (*n* 249).

‡ The *P* value indicates the probability of finding the values for the coefficient for slope as extreme as observed or more extreme, assuming that in fact no association between the results from the two assays (coefficient for slope is zero).

§ The *P* value indicates the probability of finding the values for the coefficient for slope as extreme as observed or more extreme of the absolute residuals. The residuals are computed from a regression of the difference on the averages. Data analysis based on ln-scale data.

|| Mean relative difference (bias) and LoA have been back-transformed from the natural log scale.

**Fig. 2.**
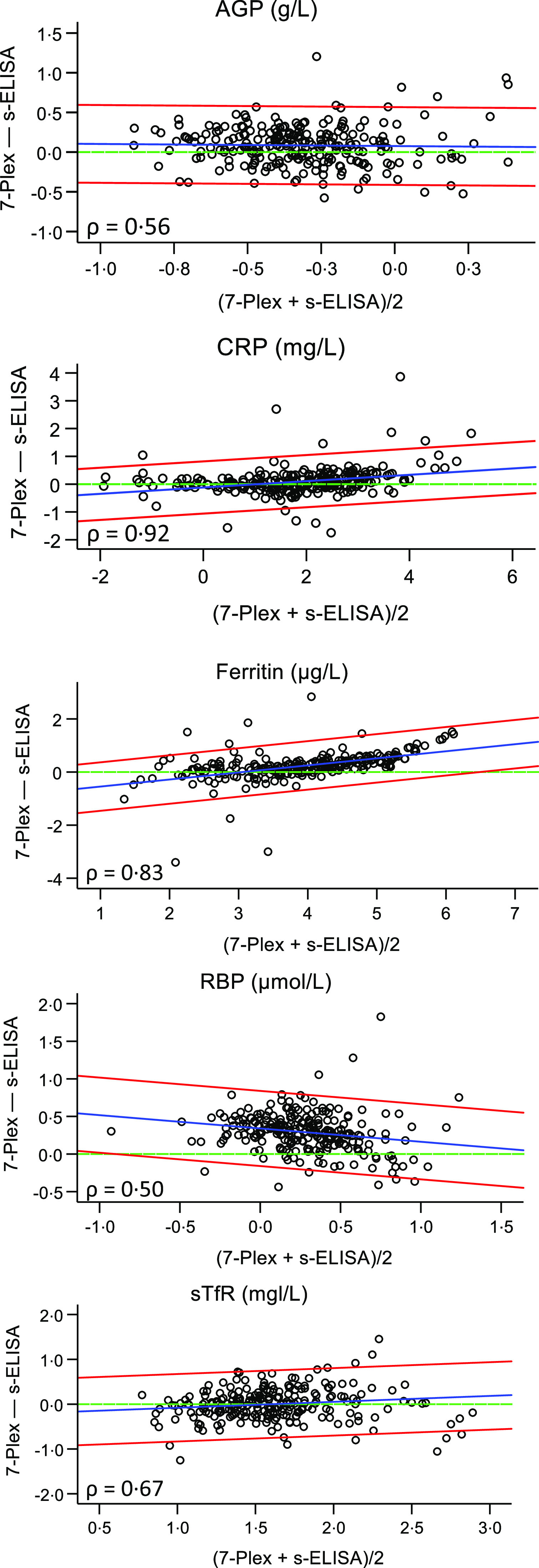
Regression-based Bland–Altman plots showing differences between results from 7-Plex and s-ELISA immunoassay (y-axes) plotted against average concentrations (x-axes). AGP, CRP, ferritin, RBP (*n* 249), and sTfR (*n* 243; see text). Differences based on log-transformed variables. Solid blue line: linear regression line indicating the non-constant difference; solid red line: 95 % limits of agreement (mean difference ± 2 sd) calculated from linear regression; dotted green line: line of identity (perfect concordance). AGP, *α*-1-acid glycoprotein; CRP, C-reactive protein; RBP, retinol-binding protein, sTfR, soluble transferrin receptor.

Visual inspection of these plots on the original scales appeared to violate more than one of the assumptions for the Bland–Altman LoA approach: either there was a relationship between the difference and the mean (non-constant difference), or the assumption of homoscedastic variance (non-constant variance) was violated. Visual inspection of the Bland–Altman plots showed that, of the five analytes, the non-constant difference for AGP and sTfR improved the most (online Supplementary Table 2). In Fig. 2, good agreement for CRP, ferritin and sTfR analyte concentrations as well as upward trend, that is, the difference between the measures is a function of the average of the measures, is shown. For all analytes but sTfR, < 5 % of the values were outside the LoA, with the majority lying above the upper limit. In addition, the variability of differences between the 7-Plex and s-ELISA measurements increased as the magnitude of their average concentration increased (Fig. 2).

## Discussion

Our results showed very strong correlations for CRP and ferritin between results obtained by Quansys Human Micronutrient Q-Plex (7-Plex) version 1.0 and the s-ELISA from VitMin Lab, and moderate concordance for CRP, whereas the other analytes performed less well. Furthermore, the 7-Plex showed largely comparable prevalence estimates with the s-ELISA, except for vitamin A deficiency.

Because neither the s-ELISA nor the 7-Plex are reference standard methods and thus not known to be superior to the other, the comparative study presented in this paper should not be seen as a method validation, but rather as an evaluation to determine if results from the 7-Plex can be used interchangeably with those from an established laboratory or assay^(7)^. It should also be noted that newer versions of Quansys Biosciences are being developed (A. Nelson, personal communication, 2021), which may result in different test performance.

To our knowledge, only one other study^(3)^ compared the 7-Plex and s-ELISA in a population at high risk of micronutrient deficiencies. In that study, agreement between assay measurements was quantified using the absolute differences between observations made using the two methods on the same subjects, and determining 95 % reference intervals for these differences^(21)^. By contrast, we used an extension of this statistical technique by using logarithmic transformation and regression approach for non-uniform differences, which is more appropriate given the distributional characteristics of and the associations between the pairs of variables investigated^(10)^.

A serum retinol (vitamin A) concentration of ≤ 0·70 μmol/l is recommended by the WHO as a marker to assess the population burden of vitamin A deficiency^(26,27)^. Its measurement requires HPLC, which is expensive, technically demanding and rarely available in developing countries. Instead, serum RBP concentration is used as a proxy measure. Because international reference material for RBP is currently unavailable, researchers have used different reference methods to evaluate the performance of RBP assays^(12)^. For example, in one study, RBP was compared with retinol as a reference assay, under the assumption that RBP and retinol circulate in plasma at one-to-one molar ratio. The RBP values obtained from the VitMin Lab s-ELISA are adjusted in the analysis to retinol equivalents by simultaneous measurement of control samples with known retinol concentration, whereas those from the Q-Plex 7 assay are unadjusted for retinol. Quansys Biosciences encourages the user to make their own inferences of the generated RBP values and advises that care should be taken when interpreting the RBP4 value as the results obtained will not provide an estimation of retinol in the sample. Thus, it is advised that RBP values are calibrated by determining retinol concentration with HPLC on a subsample of the same blood samples (A. Nelson, Quansys Biosciences, personal communication, 2021).

Calibration of RBP concentration to retinol concentration and subsequent dichotomisation of RBP concentration to values ≤ 0·70 μmol/l is problematic for two reasons. First, surveys in humans indicate that the molar concentration of retinol in serum can differ from that of RBP so that the molar ratio can differ from 1:1 depending on inflammation, protein-energy malnutrition, obesity, vitamin A status, Fe status and pregnancy^(28)^. Second, the selection of a cut point of 0·70 μmol/l for RBP concentrations can lead to biased estimates of the prevalence of vitamin A deficiency as defined by serum retinol concentration of ≤ 0·70 μmol/l^(18)^. Thus, we recommend that RBP concentrations are measured and reported without prior calibration to retinol concentration, and that cut points for dichotomisation of RBP values are selected depending on diagnostic aims of the study. In a study among Kenyan children, it was also shown that the diagnostic performance of RBP concentration in assessing vitamin A deficiency is good, but it can be improved by adding serum transthyretin concentration^(18)^. Further studies are needed to confirm this finding, with a view to potentially incorporate transthyretin as a target marker into multiplex micronutrient assays. For more extensive discussion of these issues, we refer to a previous paper^(18)^.

Similar to our results, the NiMaNu cohort study^(3)^ also found strong relationships and good agreement for CRP when comparing the 7-Plex with s-ELISA in pregnant Nigerian women, as well as poor agreement for elevated AGP. Contrary to our study, the NiMaNu cohort study found strong correlations and good agreement between methods for AGP, ferritin, RBP and sTfR^(3)^. We found good agreement between the two ELISA methods within lower concentration ranges. In our study, the discrepancies in values were at the higher end of the distributions. We, however, observed that all analyte concentrations in our sample were in the upper range of what was reported NiMaNu cohort study. This might be due to the difference in pregnancy stages of the sampled women. Pregnant women (*n* 206) included in the comparison study of the 7-Plex and s-ELISA were randomly selected from the original NiMaNu study sample pool of 654 plasma samples, and majority of these women were mostly in the third trimester^(3,29)^. Analyte concentrations are known to be lowered by physiological haemodilution during the progression of pregnancy^(30)^.

There can be large differences among sTfR assays in the cut-offs used to define Fe-deficient erythropoiesis that is due to a lack of a common reference material, differences between antibodies used in various assays^(2,3,31)^, so that the results of different assays are not directly comparable^(7,9)^.

A factor that could have affected our results was the extra freeze–thaw cycle that the s-ELISA samples had to undergo before analysis. However, Esmaeili *et al.* (2018) demonstrated that the 7-Plex assay has good stability up until five freeze–thaw cycles for all five analytes. In addition, Erhardt *et al.* (2004) reported that, except for sTfR, undiluted serum samples can undergo several freeze–thaw cycles without marked changes in analyte concentrations.

Additionally, we also observed high CV for the 7-Plex assay. This could have been due to manual pipetting into plate wells. In a recent 7-Plex cross-lab analysis, the equipment in a laboratory was found to be a source of imprecision^(12)^. Another limitation of our study was that acceptable values for the LoA (95 % of the differences to lie between ± 2 sd) were not established *a priori* ^(20)^.

We found that compared with other comparison study results of the 7-Plex that used plasma samples from pregnant Nigerian women, there was a lack of high concordance in all five analytes in our study^(3)^. This could have been due to our use of serum samples. It has been suggested that 7-Plex results may be due to different sample preparation, that is, the use of serum instead of plasma samples^(7)^. This is further corroborated by a report that EDTA plasma produced 74 % higher Q-Plex sTfR concentrations compared with serum. However, although stated by Quansys Biosciences that the assays are accurate for measuring micronutrients in both serum and plasma, future work could investigate whether the difference in sample preparation affect results.

In conclusion, values observed for all five analytes from the 7-Plex were within the expected ranges at community level in low- and middle-income countries. Except for CRP concentrations, however, the 7-Plex assay gave consistently higher readings than the s-ELISA and the difference between methods increased as the serum concentrations rose. Thus, the 7-Plex assay cannot be used interchangeably with the s-ELISA method. We concur with the manufacturer and several earlier studies that the 7-Plex should be used only as a research or as a community surveillance tool^(12)^ and not for clinical diagnostic purposes in individuals.

## Supplementary material

For supplementary material accompanying this paper visit https://doi.org/10.1017/S0007114523001782. click here to view supplementary material
