# Supplementary material for: Comparison of test performance of two commonly used multiplex assays to measure micronutrient and inflammatory markers in serum: results from a survey among pregnant women in South Africa
Source: Br J Nutr. 2023 Aug 10;131(2):248–55. doi: 10.1017/S0007114523001782 (PMC10751947; doi:10.1017/S0007114523001782)
Supplement: Supplementary file 1 [file S0007114523001782sup001.docx]

**Comparison of Quansys Q-Plex™ Human Micronutrient (7-Plex) with s-ELISA in assessing biochemical indicators in pregnant women living in South Africa.**

*Chimhashu et al 2023*

Supplementary tables


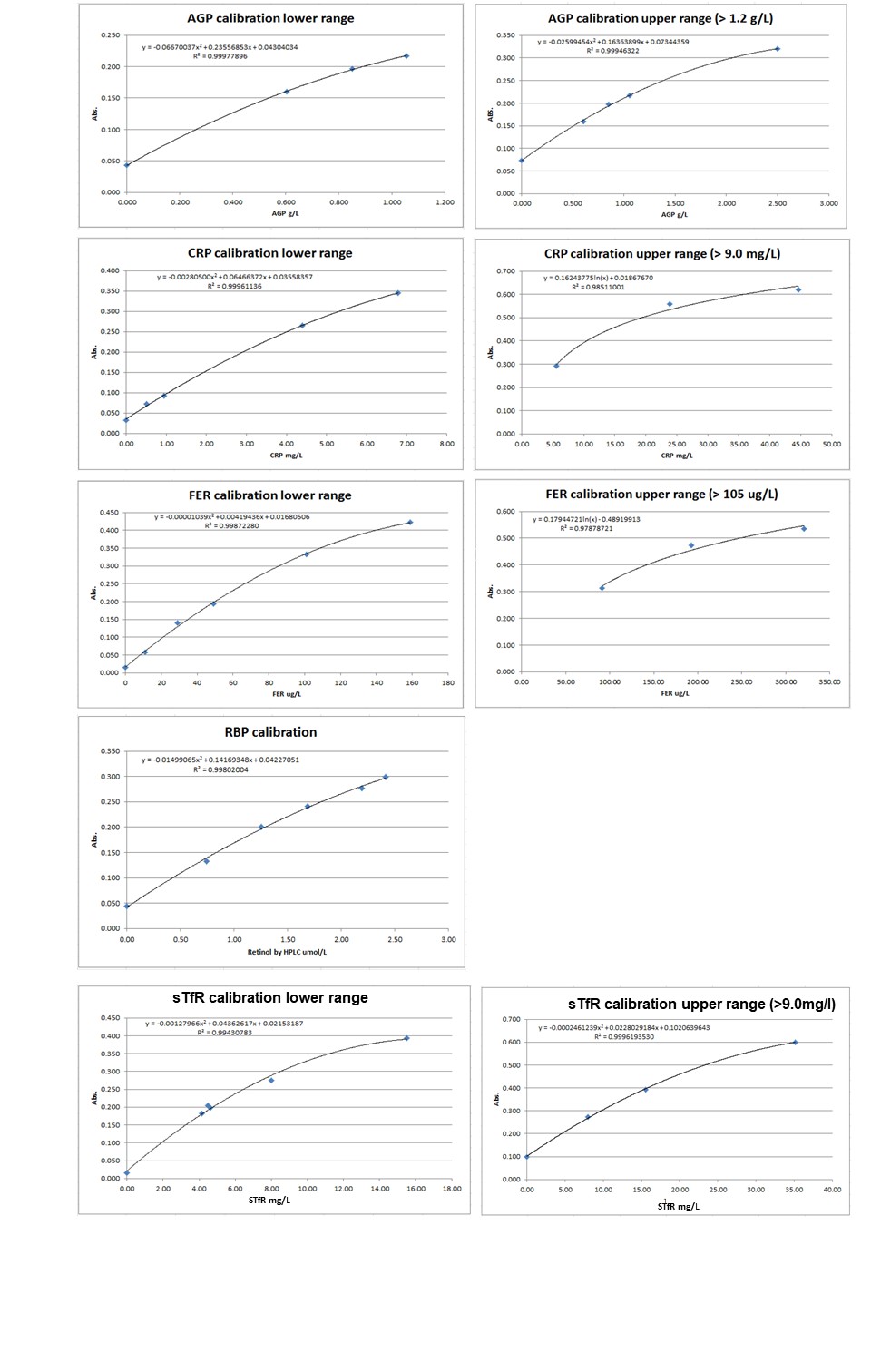


**Supplementary Figure 1.** Calibration curves from the VitMin Lab lab s-ELISA assays (lower and upper ranges); Calibration curves derived from commercially available serum control material (Bio-Rad Liquichek Immunology Control). Abbreviations: AGP, α-1-acid-glycoprotein; CRP, C-reactive protein; LOQ, limit of detection; Fer, Ferritin; RBP, retinol-binding protein; sTfR, soluble transferrin receptor


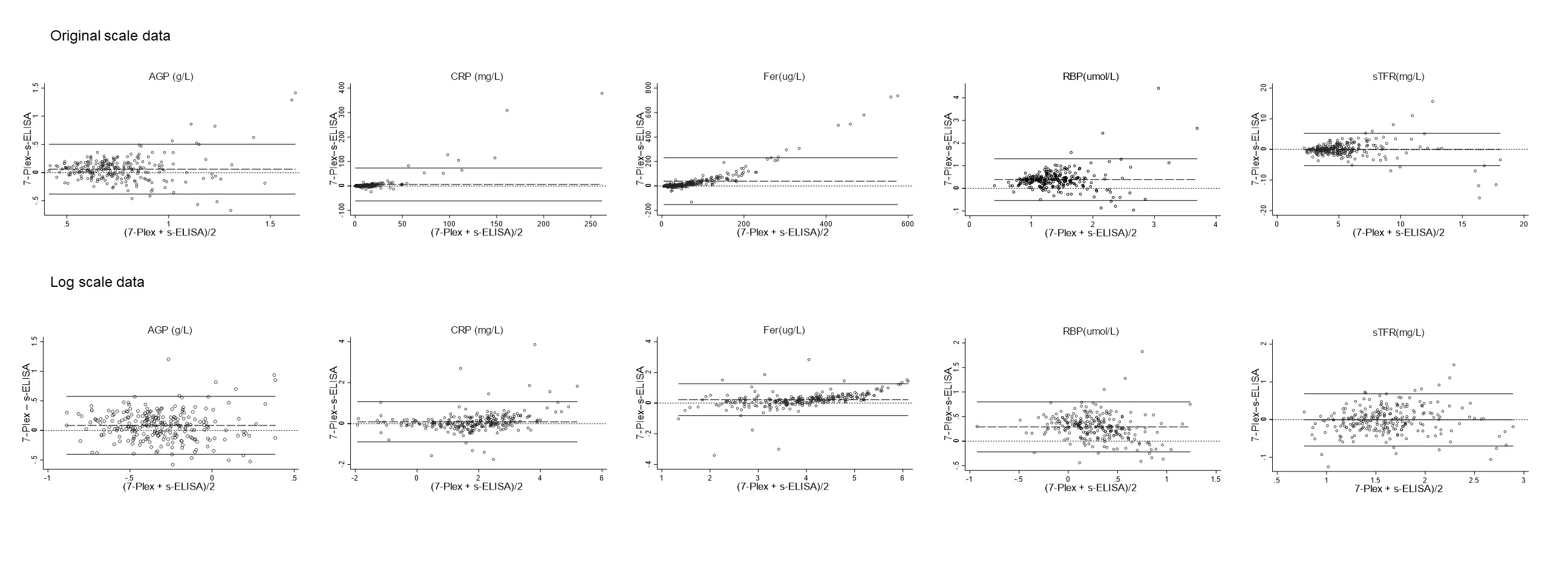


**Supplementary Figure 2** illustrates Bland-Altman plots showing the concentration difference between the 7-Plex and the s-ELISA immunoassay results (y-axes) plotted against the average concentration (x-axes) on the original and log scale. AGP, α-1-acid glycoprotein; CRP, C-reactive protein; RBP, retinol binding protein, sTfR, soluble transferrin receptor. AGP, CRP, ferritin, RBP: n=249; and sTfR: n=243 (see text). The dashed line: linear regression line represents the assessment of non-constant difference line. The solid line: limits of agreement (mean difference ± 2 SD) calculated from the linear regression. Dotted horizontal line: line of identity (perfect concordance)
